# Progress in Electrode Materials for the Detection of Nitrofurazone and Nitrofurantoin

**DOI:** 10.3390/bios15080482

**Published:** 2025-07-24

**Authors:** Mohammad Aslam, Saood Ali, Khursheed Ahmad, Danishuddin

**Affiliations:** 1 School of Chemical Engineering, Yeungnam University, Gyeongsan 38541, Republic of Korea; 2 School of Mechanical Engineering, Yeungnam University, Gyeongsan 38541, Republic of Korea; 3 Discipline of Chemistry, Indian Institute of Technology, Indore 453552, India; 4 Department of Biotechnology, Yeungnam University, Gyeongsan 38541, Republic of Korea

**Keywords:** electrode materials, nitrofurazone, nitrofurantoin, electrochemical sensors

## Abstract

Recently, it has been found that electrochemical sensing technology is one of the significant approaches for the monitoring of toxic and hazardous substances in food and the environment. Nitrofurazone (NFZ) and nitrofurantoin (NFT) possess a hazardous influence on the environment, aquatic life, and human health. Thus, various advanced materials such as graphene, carbon nanotubes, metal oxides, MXenes, layered double hydroxides (LDHs), polymers, metal–organic frameworks (MOFs), metal-based composites, etc. are widely used for the development of nitrofurazone and nitrofurantoin sensors. This review article summarizes the progress in the fabrication of electrode materials for nitrofurazone and nitrofurantoin sensing applications. The performance of the various electrode materials for nitrofurazone and nitrofurantoin monitoring are discussed. Various electrochemical sensing techniques such as square wave voltammetry (SWV), differential pulse voltammetry (DPV), linear sweep voltammetry (LSV), amperometry (AMP), cyclic voltammetry (CV), and chronoamperometry (CA) are discussed for the determination of NFZ and NFT. It is observed that DPV, SWV, and AMP/CA are more sensitive techniques compared to LSV and CV. The challenges, future perspectives, and limitations of NFZ and NFT sensors are also discussed. It is believed that present article may be useful for electrochemists as well materials scientists who are working to design electrode materials for electrochemical sensing applications.

## 1. Introduction

In the present scenario, environmental pollution is a major concern [1]. Pesticides, fungicides, pharmaceutical drugs, and toxic chemicals are released from various industries and may pollute the ground water and environment, which can significantly affect human health and aquatic life [2,3,4]. In particular, antibiotics may enter wastewater systems, greatly affect marine life, and cause antibiotic pollution [5,6]. It is also understood that degradation of antibiotics is very difficult, and it can induce detrimental effects on the environment [7]. Antibiotics are widely utilized to treat diseases, and improve productivity, therefore leading to the contamination of food products [8,9]. Antibiotic pollution may be highly associated with antibiotic resistance, which is increasing risks for human health and diseases [10,11]. In particular, the nitrofuran family of antibiotics such as nitrofurazone (NFZ) and nitrofurantoin (NFT) are extensively used for the treatment of microbial, bacterial, and protozoan infections in veterinary and farm animals [12,13]. The utilization of such substances has been limited or prohibited due to their carcinogenic effects [14]. Thus, monitoring of NFZ and NFT would be of great significance to reduce the health risks and environmental pollution due to the exposure of NFZ and NFT.

Several methods such as ultraviolet–visible (UV-vis) spectroscopy, Raman spectroscopy, fluorescence spectroscopy, and mass spectrometry have been utilized for the determination of antibiotics [15,16,17,18,19]. Unfortunately, these conventional approaches are time consuming techniques and need expensive instruments and large areas for installation. Thus, a low cost, simple, highly sensitive, selective, and stable approach should be developed for the determination of NFZ and NFT. The electrochemical sensing technique has attracted electrochemists and materials scientists due to its simplicity, high selectivity, and cost-effectiveness [20]. In the past few years, a large number of materials/electrocatalysts were prepared for the construction of electrochemical sensors for the monitoring of NFZ and NFT.

In this review article, we summarize the progress in the fabrication of NFZ and NFT sensors using various sensing techniques. The role of various electrode materials such as metal oxides, polymers, carbon derivatives, metal–organic frameworks (MOFs), layered double hydroxides (LDH), MXenes, and metals such as silver or gold nanoparticles (Ag or Au NPs) are discussed for electrochemical sensing applications.

## 2. Nitrofurazone (NFZ) Sensors

### 2.1. Metal–Organic Framework (MOF)-Based Materials for NFZ Sensing

**Scheme 1:**
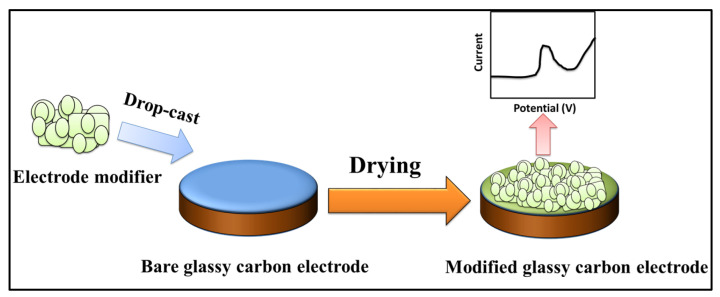
Schematic illustration of the fabrication of electrodes for electrochemical sensing applications.

It is understood that the electrochemical sensing approach involves three electrode assemblies, which are connected to a computer-controlled potentiostat system. These three electrodes can be classified as a working electrode (e.g., glassy carbon electrode (GCE), carbon paste electrode (CPE), and screen-printed carbon electrode (CPE)), reference electrode (silver/silver chloride (Ag/AgCl)), and counter electrode (platinum (Pt) electrode). The working electrode does not have sufficient catalytic activity for electrochemical sensing applications. Thus, the active area of the GCE needs to be modified with electrocatalysts using drop-casting method. The electrode modification is illustrated in Scheme 1.

**Figure 1:**
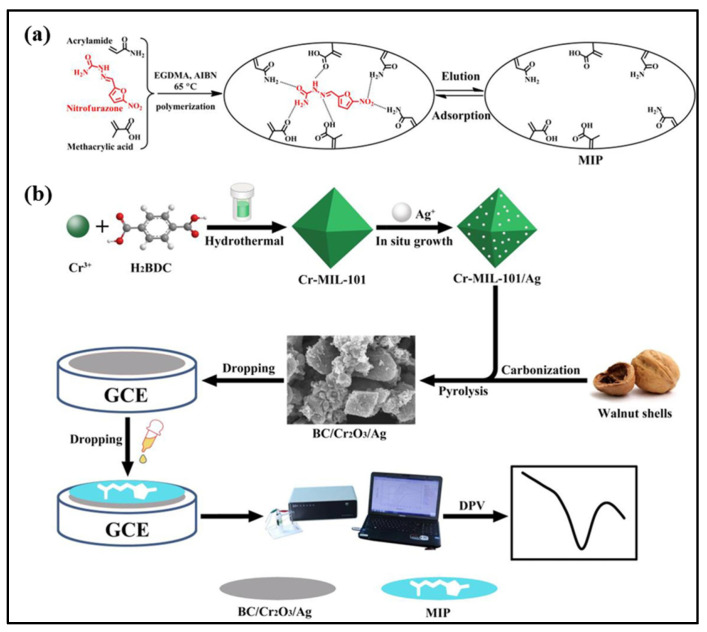
(**a**) Schematic graph shows the synthesis of MIP via precipitation polymerization method. (**b**) Schematic representation of the preparation of BC/Cr_2_O_3_/Ag and surface modification of GCE for the detection of NFZ. Reproduced with permission [23].

Previously, numerous electrode materials were extensively studied as electrocatalysts for the determination of NFZ. Metal–organic frameworks (MOFs) are well known for their high surface area and porosity, which suggests their potential for adsorption, energy storage, and electrochemical applications. It would be of great significance to explore MOF-based materials for electrochemical sensing applications. In this context, Gan et al. [21] proposed a novel electrochemical sensor for NFZ detection by modifying the surface of the GCE electrode with chromium (III) terephthalate MOF (MIL-101) hierarchical hollow cages. The SEM and transmission electron microscopy (TEM) analysis revealed the presence of a hollow structure of MIL-101, whereas X-ray diffraction (XRD) confirmed the formation of the MIL-101 with phase purity. The EIS studies indicated that a hollow MIL-101-modified GCE has improved electrical conductivity compared to the MIL-101-modified GCE. The CV studies also showed that NFZ detection with a hollow MIL-101-modified GCE is an adsorption-controlled process. The differential pulse voltammetry (DPV) analysis was conducted to further determine the effects of concentration of NFZ on the electrochemical sensing performance of the hollow MIL-101-modified GCE. The DPV studies revealed that the current response linearly increased with an increasing NFZ concentration, with an LDR of 0.030 to 55 µM and an LD of 10 nM. The authors also found that the hollow MIL-101-modified GCE has good selectivity, stability, reproducibility, and real sample recovery for NFZ detection. The excellent selectivity of the hollow MIL-101-modified GCE for NFZ detection in the presence of various interfering substances, including Sn(II), Mn(II), Mg(II), Sr(II), Ca(II), Co(II), Cr(III), Pb(II), Ni(II), Zn(II), Al(III), aspartic acid, glycine, ascorbic acid, urea, and glucose, suggests its potential for practical applications. Wang et al. [22] prepared a zirconium (Zr)-based MOF (DUT-67)/tubular polypyrrole (T-PPY) composite using the solvothermal-assisted method. The SEM analysis revealed that the DUT-67 MOF is strongly cross-linked with T-PPY and a cross-linked network was formed, which may reduce the agglomeration of DUT-67 crystals. The nitrogen (N_2_) adsorption–desorption isotherm of the prepared DUT-67/T-PPY-2 composite displayed a specific surface area of 336.76 m^2^/g, with an improved pore size of 1.62 nm, which can significantly accelerate the mass transfer. The DPV studies also showed that the DUT-67/T-PPY-2 composite-modified GCE can detect NFZ with an LD of 8.7 µM and LDR of 9.08 to 354.08 and 354.08 to 1004.4 µM. The milk and lake water sample-based studies further suggested the potential of the DUT-67/T-PPY-2 composite-modified GCE for NFZ detection in real samples. Cheng et al. [23] synthesized an MO- derived chromium oxide (Cr_2_O_3_)/silver nanoparticle (NP)/biomass carbon (BC) composite. The preparation of the molecularly imprinted polymer (MIP) is shown in Figure 1a.

**Figure 2:**
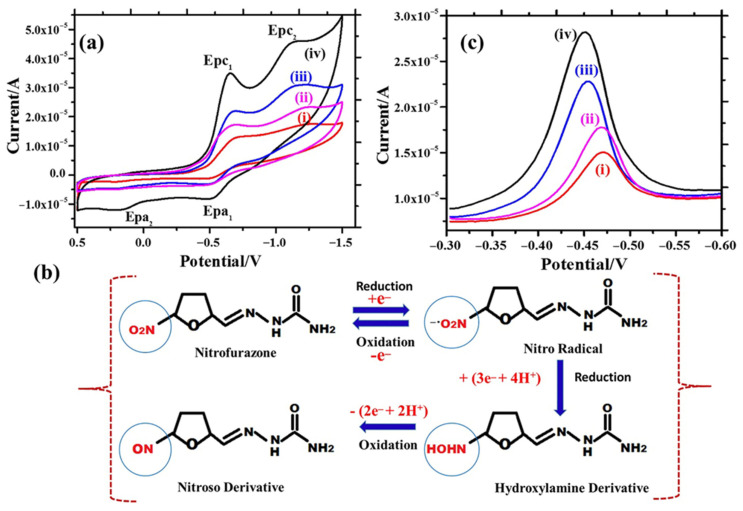
(**a**) CV curves of the (i) SPCE, (ii) UiO-66-NH_2_-modified SPCE, (iii) Au NP-modified SPCE, and (iv) Au NP/UiO-66-NH_2_-modified SPCE for NFZ detection. (**b**) Mechanism for electrochemical detection of NFZ. (**c**) DPV curves of the (i) SPCE, (ii) UiO-66-NH_2_-modified SPCE, (iii) Au NP-modified SPCE, and (iv) Au NP/UiO-66-NH_2_-modified SPCE for NFZ detection (15 µM; pH = 7.4 and 0.1 M PBS). Reproduced with permission [24].

The MIP-based BC/Cr_2_O_3_/Ag-modified GCE was constructed for the determination of NFZ. The fabrication of the NFZ sensor and its mechanism is illustrated in Figure 1b. The BC/Cr_2_O_3_/Ag/MIP/GCE exhibited an LD of 3 nM and LDR of 5 nM to 10 µM using the DPV method. The authors also observed that the proposed BC/Cr_2_O_3_/Ag/MIP/GCE has decent stability, reproducibility, and recovery in biological fluids for NFZ detection. The proposed sensor also shows higher selectivity in the presence of various interfering species such as dopamine hydrochloride, glucose, furostone hydrochloride, ascorbic acid, semicarbazide hydrochloride, sucrose, soluble starch, and lactic acid. Rani et al. [24] reported the synthesis of a Zr-based MOF (Zr-MOF or UiO-66-NH_2_) and combined it with gold nanoparticles (Au NPs) to fabricate the NFZ sensor using a screen-printed carbon electrode, i.e., SPCE as the working electrode. The synthesized MOF was characterized by SEM, XRD, and energy-dispersive X-ray spectroscopy (EDX) to confirm its structural, phase purity, and elemental composition. The CV curves of the SPCE, UiO-66-NH_2_-modified SPCE, Au NP-modified SPCE, and Au NP/UiO-66-NH_2_-modified SPCE for 15 µM NFZ are shown in Figure 2a. It can be seen that the Au NP/UiO-66-NH_2_-modified SPCE displayed a higher current response for NFZ detection compared to the other modified electrodes and bare SPCE. The mechanism for NFZ detection is illustrated in Figure 2b. It can be clearly observed that the first cathodic peak, i.e., E_pc1_, may be ascribed to the one electron reduction of the NO_2_ group of NFZ to the NO_2_ radical. The free NO_2_ radical is reduced (E_pc2_) with the involvement of three electrons and four protons to form the hydroxylamine derivative. The other two peaks may be ascribed to the oxidation of the nitro radical to NFZ (E_pa1_) and the hydroxylamine derivative to a nitroso derivative (E_pa2_), as shown in Figure 2b. The DPV curves of the SPCE, UiO-66-NH_2_-modified SPCE, Au NP-modified SPCE, and Au NP/UiO-66-NH_2_-modified SPCE for 15 µM NFZ are shown in Figure 2c. The DPV results also indicated that the Au NP/UiO-66-NH_2_-modified SPCE has improved catalytic properties for the determination of 15 µM NFZ. The Au NP/UiO-66-NH_2_-modified SPCE exhibits an LD of 3 nM and LDR of 1 × 10^−8^ to 5 × 10^−5^ M for NFZ detection, with an acceptable recovery of 93.3% to 97.84% in a lake water sample.

Leelasree et al. [25] proposed a novel cobalt (Co)-based two-dimensional (2D) MOF as the sensing layer for the construction of an NFZ electrochemical sensor. The surface morphology of the synthesized 2D Co MOF was characterized by the SEM technique. The SEM results show that Co MOF crystals are block-shaped and havie size of 50 mm to 250 mm. Furthermore, the 2D Co MOF-modified GCE exhibited reasonable electrochemical performance for NFZ detection via CV, square wave voltammetry (SWV), and amperometry methods. The authors obtained an LD of 40 nM and excellent selectivity towards the monitoring of NFZ. The MOFs possess a larger surface area and pore size, which makes them a promising catalyst for electrochemical sensing applications. The above-mentioned reports show that MOF-based electrode materials are promising sensing layers for the construction of NFZ electrochemical sensors.

### 2.2. Carbon Derivative-Based Materials for NFZ Sensors

Carbon derivatives such as graphene, carbon nanotubes (CNTs), and graphitic carbon nitride (g-C_3_N_4_) are widely used for electrochemical sensing applications. It is understood that functionalization of carbon-based materials may exhibit enhanced electrocatalytic properties for redox reactions. He et al. [26] adopted carboxyl (COOH) group-functionalized multi-walled carbon nanotubes (MWCNTs) as sensing materials for the construction of NFZ sensors. In this context, these authors used sulfuric acid (H_2_SO_4_)/nitric acid (HNO_3_) (ratio = 3:1) to functionalize the MWCNTs and drop-casted it on the surface of a glassy carbon electrode (GCE). The functionalization was confirmed by Fourier Transform infrared spectroscopy (FTIR), while SEM results indicated that the COOH-MWCNTs has significantly changed structure. It was also observed that the COOH-MWCNTs has a larger specific surface area, which makes it a promising material for sensing applications. Electrochemical impedance spectroscopy (EIS) was also explored to check the conductive properties of the COOH-MWCNT-modified GCE in the presence of a 10 mM [Fe(CN)_6_]^3-/4-^ redox system. The obtained results demonstrated that the COOH-MWCNTs have a relatively higher electrical conductivity compared to the pristine MWCNT-modified GCE. This is revealing that the presence of COOH may enhance the electrical conductivity of the COOH-MWCNT-modified GCE. The CV and amperometry were explored for the sensing of NFZ. The authors optimized the pH conditions for the improved sensing of NFZ. The COOH-MWCNT-modified GCE displayed an LD of 0.188 µM, with successful detection of NFZ in real samples. Brito et al. [27] also explored the potential of hemin complex-modified MWCNTs as the sensing layer for the development of NFZ sensors. The hemin-functionalized COOH-MWCNTs displayed interesting electrocatalytic properties. In another report, Cai et al. [28] proposed the construction of a functionalized polyoxometalate/graphene-based electrode as the NFZ sensor. The authors used the layer-by-layer assembly method for the fabrication of NFZ sensors, and its electrochemical activity for NFZ sensing was evaluated by employing voltammetry and amperometry methods. It was found that the proposed sensor displayed an LD of 0.4548 μM, with reasonably good stability, reproducibility, and recovery in crayfish samples using the spike addition method. This proposed NFZ sensor was also selective for NFZ sensing in the presence of various electro-active species (glycine, semicarbazide hydrochloride, K^+^, Na^+^, Cl^−^, phenylalanine, lysine, cysteine, ascorbic acid, Zn^2+^, CO_3_^2–^, NO_3_^–^, SO_4_^2−^ and glucose, Ca^2+^ Mg^2+^, and Cu^2+^). The reduced graphene oxide (rGO) and Au NPs can be sequentially modified onto the surface of the GCE to improve the effective surface area and electron transfer capability of the GCE. The authors proposed the fabrication of an MIP electrochemical sensor using Au NPs/rGO as the sensing material for NFZ detection using DPV analysis [29]. The proposed electrochemical sensor delivered an LD of 0.18 nM with an LDR of 5 to 1000 nM for the sensing of NFZ with excellent repeatability (relative standard deviation (RSD) = 1.17%) and stability. Jiang et al. [30] synthesized laser-induced graphene for sensing applications. The electrochemical investigations revealed that an engraved graphene array with a 50% laser power, i.e., the LIG-50 array, has higher electrocatalytic properties and higher stability for NFZ detection. The authors obtained an LD of 35 nM and LDR of 0.2 to 8 µM with reasonable recovery in fish meat samples. The improved electrochemical activity of the LIG-50 array was attributed to the three-dimensional (3D) porous structure. The above-mentioned studies revealed that graphene and MWCNT-based materials have promising features for NFZ sensing applications.

### 2.3. Metal Oxide-Based Materials for NFZ Sensors

**Figure 3:**
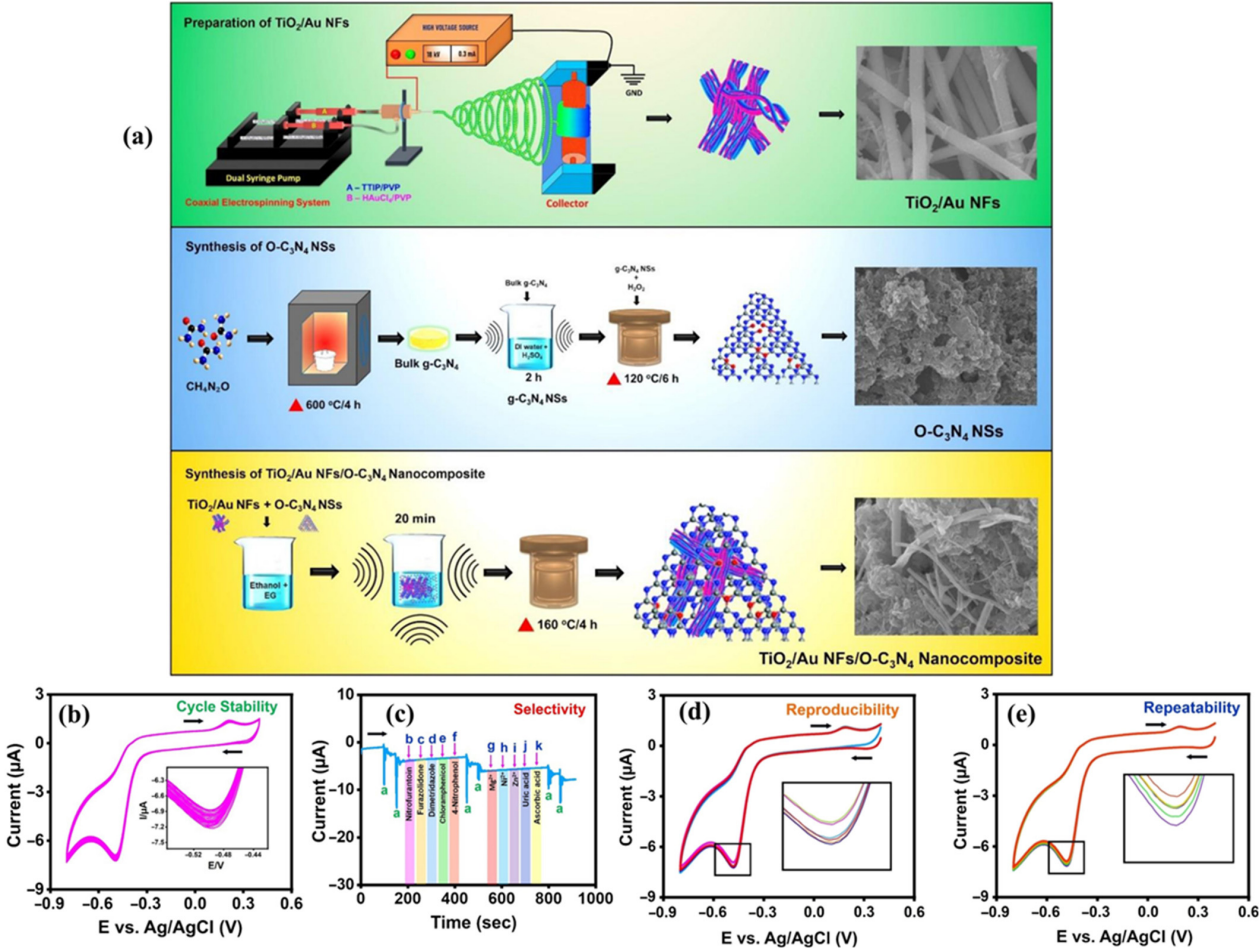
(**a**) Schematic graph shows the fabrication of electrode material (TiO_2_/Au-NFs/O-C_3_N_4_). (**b**) Cyclic stability, (**c**) selectivity, (**d**) reproducibility, and (**e**) repeatability. Reproduced with permission [31].

Metal oxides are the stable material for long-term applications and their nanostructured characteristics make them a suitable electrocatalyst for optoelectronic applications. Titanium dioxide (TiO_2_) is one of the transition metal oxides which has high electron mobility, decent biocompatibility, low cost, and excellent stability. Kokulnathan et al. [31] fabricated a TiO_2_ nanofiber (NF)/Au NP/O-doped g-C_3_N_4_ nanosheet (TiO_2_/Au-NFs/O-C_3_N_4_) composite and deposited it on the surface of the GCE. The synthetic process is described in Figure 3a. The TiO_2_/Au-NF/O-C_3_N_4_-coated GCE exhibited a good LDR of 0.008 µM to 105 µM, LD of 1 nM, cyclic stability (Figure 3b), selectivity (Figure 3c), reproducibility (Figure 3d), and repeatability (Figure 3e). The introduction of O in the g-C_3_N_4_ matrix may enhance active sites, catalytic stability, improve the interlayer distance, and accelerate the redox reactions for the detection of NFZ. The constructed electrode material, i.e., TiO_2_/Au-NFs/O-C_3_N_4_, exhibited a larger surface area and promoted ionic/electron transportation and surface functional groups, which enhance the detection of NFZ. Therefore, the TiO_2_/Au-NF/O-C_3_N_4_-coated GCE is a promising working electrode for NFZ detection.

**Figure 4:**
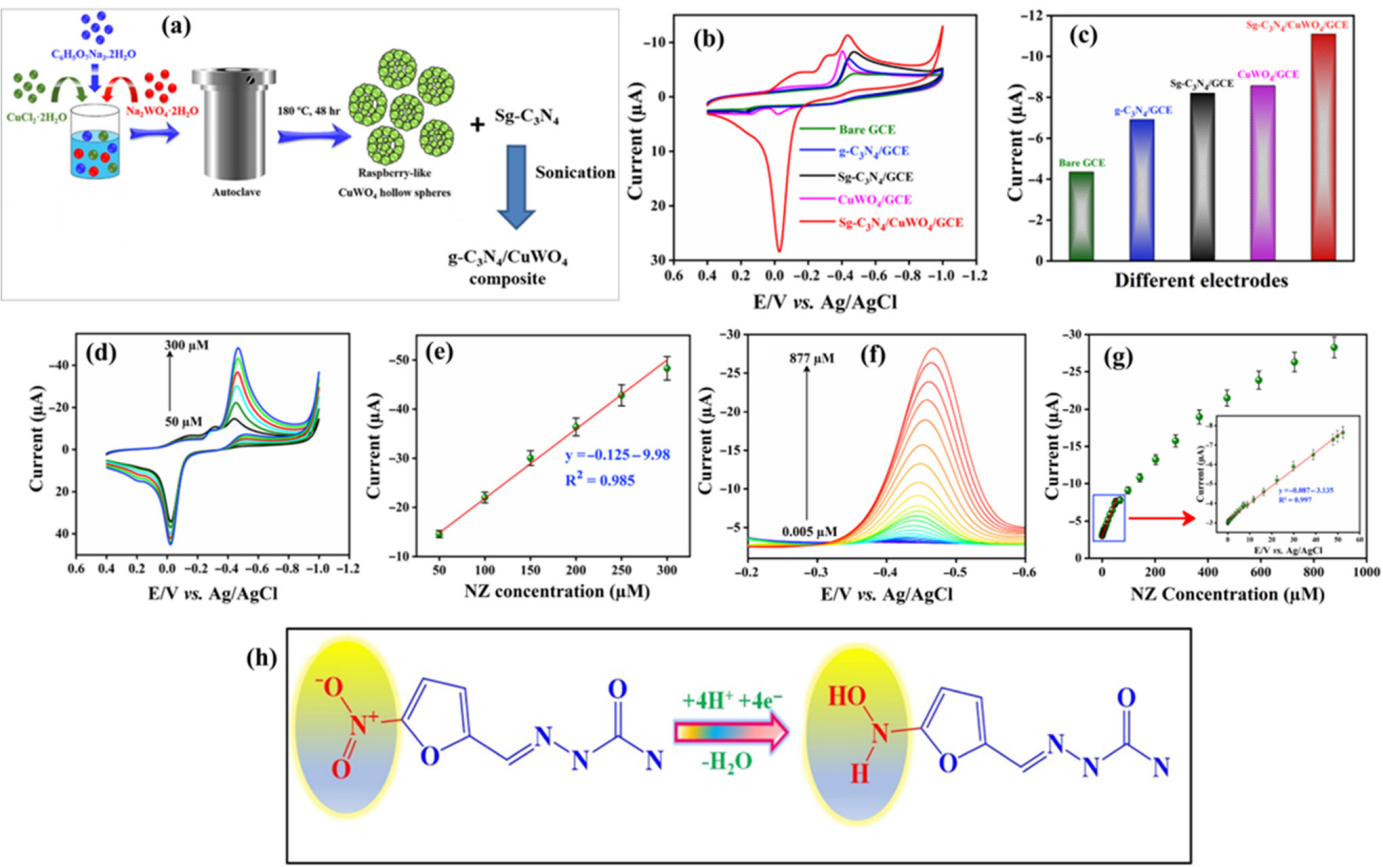
(**a**) Schematic picture represents the fabrication of Sg-C_3_N_4_/CuWO_4_. (**b**) CV curves and (**c**) corresponding bar diagram of the different electrodes for NFZ sensing. (**d**) CV curves of Sg-C_3_N_4_/CuWO_4_/GCE for various concentrations of NFZ and (**e**) corresponding calibration plot. (**f**) DPV curves of Sg-C_3_N_4_/CuWO_4_/GCE for different concentrations of NFZ and (**g**) corresponding calibration curve. (**h**) Sensing mechanism for NFZ detection. Reproduced with permission [33].

In another study [32], a g-C_3_N_4_/gadolinium molybdate (g-C_3_N_4_/Gd_2_MoO_6_) composite was synthesized using co-precipitation and sonication methods. The g-C_3_N_4_/Gd_2_MoO_6_ was deposited on the surface of the GCE for the sensing of NFZ. It was observed that the g-C_3_N_4_/Gd_2_MoO_6_-modified GCE has improved electrocatalytic properties compared to the bare GCE, and g-C_3_N_4_ and Gd_2_MoO_6_-modified electrodes. Therefore, it was observed that g-C_3_N_4_/Gd_2_MoO_6_GCE can detect NFZ with an LD of 6 nM and LDR of 0.02 µM to 2000 µM under the optimized experimental conditions. Finally, the proposed NFZ sensor was also selective with acceptable recovery in real water samples. Anupriya et al. [33] reported the fabrication of a Sg-C_3_N_4_/CuWO_4_ composite by employing a benign ultrasonication method. The authors stated that Sg-C_3_N4 and CuWO_4_ exhibited enhanced adhesive interactions and hydrodynamic shear forces during the sonication treatment. Thus, ultrasonication treatment developed interactions between CuWO_4_ and a layered Sg-C_3_N_4_ and Sg-C_3_N_4_/CuWO_4_ composite was formed. The preparation of the Sg-C_3_N_4_/CuWO_4_ composite is presented in Figure 4a. The SEM results indicated that Sg-C_3_N_4_ has a flake-like structure, and CuWO_4_ has hollow spheres. The Sg-C_3_N_4_/CuWO_4_ composite was deposited on the GCE and its electrochemical activity with other electrodes were examined in the presence of NFZ using the CV method.

The obtained CV results for the different modified electrodes are displayed in Figure 4b, and it was observed that the Sg-C_3_N_4_/CuWO_4_ composite-modified GCE has a higher current response compared to the other electrode (Figure 4c). The effects of different NFZ concentrations were also studied using CV and DPV methods, as shown in Figure 4d,e and Figure 4f,g, respectively. It was clearly observed that the current response of the Sg-C_3_N_4_/CuWO_4_ composite-modified GCE linearly increases with an increasing concentration of NFZ (Figure 4e,g). The sensing mechanism for NFZ detection is illustrated in Figure 4h. In brief, it can be seen that electrochemical determination of NFZ involves the reduction of nitro (NO_2_) groups. During electrochemical detection of NFZ, the -NO_2_ group transformed to a hydroxylamine (-NHOH) group, as shown in Figure 4h. The transformation of the –NO_2_ group to the –NHOH group can be explained as given below:

-NO_2_ + 4H^+^ + 4e- → -NHOH + H_2_O. (1)

It was found that the Sg-C_3_N_4_/CuWO_4_ composite-modified GCE can be used for the detection of NFZ in human urine and serum samples. The Sg-C_3_N_4_/CuWO_4_ composite-modified GCE also delivered an LD of 3 nM with excellent repeatability, selectivity, reproducibility, and long-term storage stability. The presence of the synergism between Sg-C_3_N_4_ and CuWO_4_ enhanced the electrochemical performance of the Sg-C_3_N_4_/CuWO_4_/GCE and may be explored for practical applications. In a previous report [34], a neodymium oxide@titanium carbide (NdO@TC) composite was fabricated by using the ultrasonication method. It was observed that NdO has a nanoflake-like morphology, whereas exfoliated TC exhibited a micro sheet-like structure. The NdO@TC was cast on the surface of the GCE and its electrochemical properties for NFZ detection was determined by using CV and DPV methods. The proposed electrode showed a sensitivity of 0.1914 µA µM^−1^ cm^−2^ and LD of 2.7 nM, with an LDR of 0.01 µM to 2231 µM. Nair et al. [35] constructed a NFZ sensor by using iron titanate (FeTiO_3_) as the sensing layer. It was found that FeTiO_3_ was comprised of hexagonal nanoplates, which may exhibit improved electrocatalytic properties. Thus, these authors modified the surface area of the GCE with FeTiO_3_ and utilized it as an NFZ sensor. The improved electrochemical performance of the FeTiO_3_/GCE may be ascribed to the enhanced electrical conductivity, abundant active sites, and improved specific surface area of the FeTiO_3_.Therefore, a low LD of 2 nM, sensitivity of 0.551 µA µM^−1^ cm^−2^, and LDR of 0.01 µM to 162.2 µM were obtained for the FeTiO_3_/GCE-based NFZ sensor. Pandiyan et al. [36] developed a dysprosium ferrite (DFO) NP decorated S-g-C_3_N_4_ composite using the sonication method. The EIS studies show that the DFO/S-g-C_3_N_4_ composite-modified GCE has a higher electrochemical active surface area and improved electrical conductivity. Thus, the DFO/S-g-C_3_N_4_ composite-modified GCE demonstrated an LD of 7.1 nM, decent reproducibility, good selectivity, repeatability, and recovery in river and lake water samples. The improved conductivity and synergy effects were responsible for the enhanced sensing performance of the proposed NFZ sensor. In another study [37], crab shell carbon was also prepared at 500 °C (C-CS-500), 700 °C (C-CS-700), and 900 °C (C-CS-900) for the construction of the NFZ sensor. The C-CS-700-modified GCE exhibited better electrochemical properties compared to the C-CS-500 and C-CS-900-modified electrodes. Thus, the C-Cs-700/GCE exhibit an LD of 110 nM and LDR of 0.40 to 80 µM, with satisfactory recovery in compound cod liver oil ointment samples.

### 2.4. Other Material-Based NFZ Sensors

Rahi et al. [38] proposed the fabrication of Au nanorods (NRs) on a Au electrode surface by employing the sonoelectrodeposition approach. The authors characterized the surface morphology of the electrodeposited Au NR/Au electrode by employing SEM analysis, which revealed that the surface is covered with Au NRs. Furthermore, the fabricated Au NR/Au electrode was utilized as an NFZ sensor, and its electrochemical performance was evaluated by employing amperometry and DPV methods. The good electrocatalytic properties and presence of the NR-like surface morphology of the Au NRs enhanced the detection of NFZ, and the limit of detection of 6.51 µM and linear dynamic range of 50 to 610 µM were obtained for NFZ detection via the amperometry method. In another work, an LD of 0.18 µM with an LDR of 3 to 500 µM were observed for NFZ detection via DPV analysis. The NFZ was also detected in biological and pharmaceutical samples such as urine and serum samples using a Au NR/Au electrode. Zoubir et al. [39] fabricated silver (Ag) on graphite carbon paste (GCP) by utilizing the electrochemical method. The pH effect, number of scan cycles, electrodeposition potential, and scan rate effects were optimized, and the obtained Ag-deposited GCP was explored for NFZ detection. The Ag-deposited GCP electrode exhibited high electrical conductivity and a larger surface area, which are beneficial characteristics for any sensing material. Thus, Ag-deposited GCP electrode showed an LD of 10^−8^ M for the detection of NFZ. This sensor was also efficient for NFZ detection in urine and tap water samples, with satisfactory recovery of more than 94%. Yeh et al. [40] proposed the fabrication of a highly sensitive and selective electrochemical sensors for the monitoring of NFZ and semicarbazide. In this connection, Au-Ag was fabricated on copper (Cu) foil by employing the electrodeposition method. The CV studies show that deposition of Au-Ag was feasible and was characterized by SEM, XRD, and X-ray photoelectron spectroscopy (XPS). The XPS studies authenticated the formation of Au-Ag on Cu foil with decent phase purity. The Au-Ag-based NFZ sensor demonstrated an LDR of 1.99 to 643.49 µM, LD of 200 nM, and high selectivity, with an average recovery of 100.59% in real samples.

Lu et al. [41] proposed that integration of MIP and electrochemical sensors may exhibit excellent selectivity and stability for the determination of NFZ. In this regard, these authors reported the preparation of bifunctional monomers/zeolitic imidazolate framework (ZIF)-modified COOH-MWCNTs for the monitoring of NFZ. The authors stated that this combination may improve the low conductivity and limited number of imprinted holes in the MIP membrane. It was also found that the porous nature of the proposed composite may enhance the active surface of the electrode and provide abundant molecularly imprinted holes. Thus, improved detection of NFZ can be observed using the proposed composite-modified electrode. Therefore, an interesting LD of 6.7 × 10^−14^ M and LDR of 10^−13^ M to 10^−6^ M were obtained with reasonably good selectivity. The presence of unique holes in the fabricated electrode was the key point for the improved selectivity of the NFZ sensor. The authors also found that the proposed novel NFZ sensor has good reproducibility, stability, and satisfactory recovery in urine and water samples. Zoubir et al. [42] reported the construction of a Ag NP-modified CPE (CPE = carbon paste electrode) and evaluated its electrochemical properties for NFZ monitoring. The proposed Ag NP-modified CPE showed a larger active surface area and high electrical conductivity, which makes it a promising candidate for electrochemical sensing applications. The proposed NFZ sensor delivered good sensing performance in terms of real sample studies with satisfactory recovery in human urine, tap water, and commercial milk samples. The pH was also optimized and DPV analysis demonstrated a good LD of 12 nM and LDR of 10^−4^ M to 2 × 10^−7^ M for NFZ sensing.

**Figure 5:**
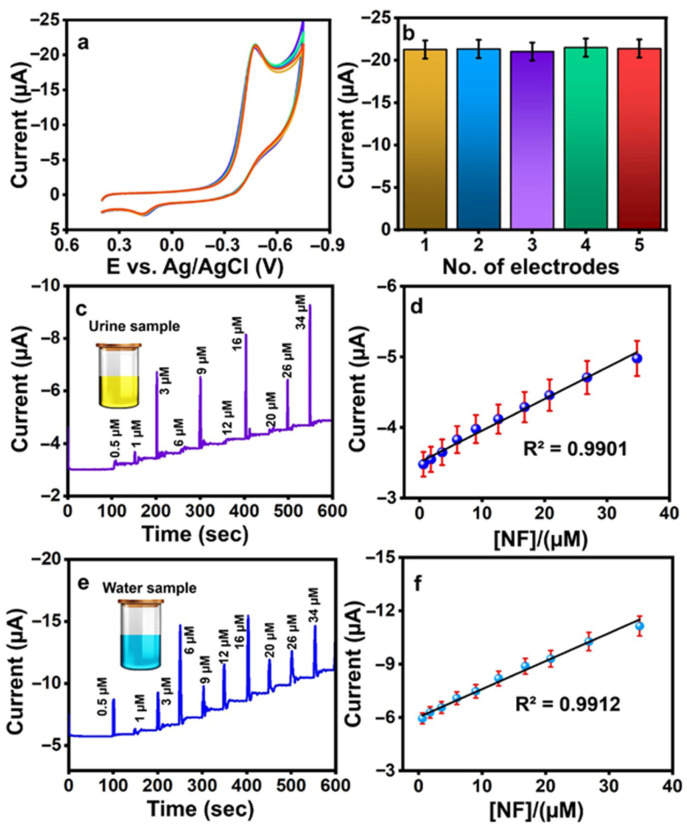
(**a**) CV curves and (**b**) corresponding bar graph for five different SnSe/h-BN-modified electrodes for NFZ sensing. Real-time sensing of SnSe/h-BN-based electrode for NFZ sensing in urine (**c**,**d**) and water (**e**,**f**) samples. Reproduced with permission [43].

Thirumalraj et al. [43] explored the potential of a layered metal chalcogenide (tin selenide = SnSe)-modified hexagonal boron nitride (h-BN) using simple strategies. The SnSe/h-BN composite-modified GCE demonstrated high electrical conductivity compared to the SnSe or h-BN-modified GCE. This may be attributed to the presence of synergistic interactions between SnSe and h-BN. CV and amperometry methods were used for the determination of NFZ. The LD of 0.34 nM and sensitivity of 1.927 µA µM^−1^ cm^−2^, with two LDRs of 0.001 µM to 12.12 µM and 15.2 µM to 342.2 µM, were observed for the constructed NFZ sensor. The proposed NFZ sensor also displayed good reproducibility, as confirmed by CV studies (Figure 5a,b). The real-time sensing performance for the SnSe/h-BN/GCE was also evaluated in presence of urine (Figure 5c) and water (Figure 5e) samples. The authors also observed that NFZ can be detected in urine and water samples with good linearity, as shown in Figure 5d,f, respectively.

This excellent electrochemical activity of the constructed electrode may be ascribed to the larger active surface area and synergism between the SnSe and h-BN materials, which significantly improved the kinetics of the electron transfer between the electrode and electrolyte interface. Yazdani et al. [44] adopted the hydrothermal method for the synthesis of nickel microparticle-covered nanoflakes (NMCNs). The NMCN-modified CPE displayed an LD of 4.3 µM and LDR of 30 µM to 570 µM, with satisfactory recovery in ointments and human serum samples. Silva et al. [45] reported disposable electrochemical sensors by using a screen-printed electrode (SPE) as the working substrate and Ag NPs/carbon quantum dots (CQDs)/CNTs as the electrode material. The proposed SPE/Ag NPs/CQDs/CNTs showed an LD of 4.6 nM and LDR of 0.008 to 15.051 µM for NFZ monitoring. This performance may be attributed to the presence of synergistic interactions and improved conductive nature of the composite material. The electrochemical sensors can be explored for the monitoring of NFZ in environmental monitoring applications. Thus, these authors also observed that SPE/Ag NPs/CQDs/CNTs have an acceptable recovery of 86.4% to 102% in river, tap, ground water, and leachate water samples via the spiked method. The authors also found that SPE/Ag NPs/CQDs/CNTs can detect NFZ in ointment M and dermatologic solution N, with a satisfactory recovery of 95.6% and 93.3%, respectively. The electrochemical parameters for the reported NFZ electrochemical sensors are compiled in Table 1.

## 3. Nitrofurantoin (NFT) Sensors

### 3.1. Carbon Derivative-Based Materials for NFT Sensing

**Figure 6:**
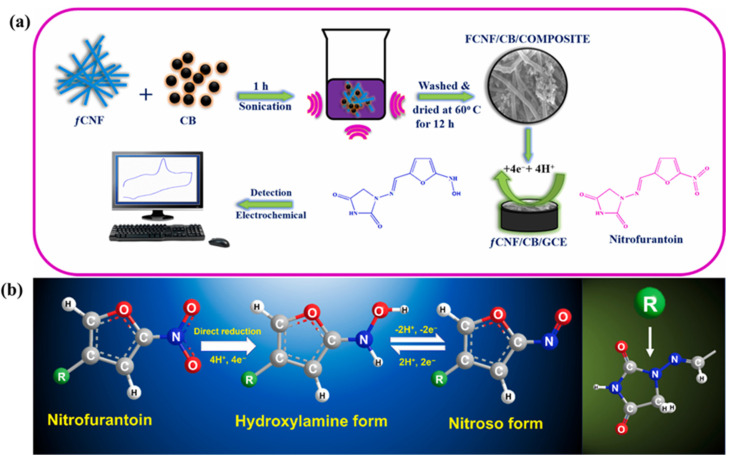
Schematic representation of the (**a**) fabrication of f-CNF/CB composite and (**b**) probable sensing mechanism for NFT detection. Reproduced with permission [49].

He et al. [46] observed that Au NPs/graphene has enhanced conductive properties, and the presence of a larger specific area may promote better electron transportation between the interface of the electrode and electrolyte interface. Thus, the Au NP/graphene-modified electrode exhibited an LD of 130 nM and two LDRs of 0.2 µM to 80 µM and 280 µM to 4800 µM for NFT monitoring using the DPV method. The DPV-based studies also revealed that the current response for the monitoring of NFT linearly increases with an increasing concentration of NFT. The ultrasonication method was also adopted by Velmurugan et al. [47] for the functionalization of MWCNTs. In this context, a chitosan hydrogel (CHI)/MWCNT composite was obtained using the ultrasonic-assisted approach. The authors also prepared rod-like hydroxyapatite NPs (HA NPs) using the hydrothermal method. Furthermore, HA NPs were incorporated with CHI/MWCNTs via the ultrasonication method. The obtained HA NP/CHI/MWCNTs were coated on the surface of the GCE and its electrochemical activity for NFT detection was performed using CV and amperometry methods. The current response of the HA NP/CHI/MWCNT/GCE was increased with an increasing concentration of NFT and these authors observed that detection of NFT is a diffusion-controlled process. The LD of 1.3 nM, LDR of 0.005 µM to 982.1 µM, sensitivity of 0.16 µA µM^−1^ cm^−2^, and high selectivity were observed, which may be due to the presence of a high surface area and synergistic effects. Hwa et al. [48] reported a simple and cost-effective sensor for the determination of NFT using a nickel iron (NiFe)/f-MWCNT composite as an efficient electrocatalyst. The NiFe/f-MWCNT composite was obtained by hydrothermal and sonication methods and TEM analysis suggested that chain-like nanospheres are anchored on f-MWCNTs without any significant agglomeration. This proposed electrode material was coated on the surface of SPCE, and electrochemical investigations revealed that NFT can be detected with an LD of 30 nM and sensitivity of 11.45 µA µM^−1^ cm^−2^. In another study [49], a functionalized carbon nanofiber/carbon black (f-CNF/CB) composite was synthesized using the simple ultrasonication method, as shown in Figure 6a. The SEM analysis revealed that CB NPs are paired with the f-CNF. It was observed that strong aggregation of the f-CNF and CB occurred due to the presence of COOH groups in the f-CNF, which can easily form the connection between f-CNF and CB. The f-CNF/CB was coated on the GCE, and the electrochemical investigation shows that NFT detection involves an adsorption-controlled process. This NFT sensor exhibits an LD of 16 nM, sensitivity of 15.25 μA µM^−1^ cm^−2^, and LDR of 0.05 µM to 104.66 µM. The improved sensing performance of the above-mentioned NFT sensor may be attributed to the presence of the synergistic effects, decent ionic conductivity, and fast transfer behavior. The NFT has a nitrofuran ring with a –NO_2_ group, which participates in electrochemical sensing of NFT. The –NO_2_ group was reduced to a –NHOH group, and this process can be explained below as follows:

-NO_2_ + 4H^+^ +4e^-^ → -NHOH. (2)

The other step can be explained below:

-NHOH ↔ NO + 2H^+^ +2e^-^. (3)

The transformation of –NO_2_ to –NHOH is an expected irreversible process, whereas –NHOH to NO conversion is a reversible process. This mechanism is illustrated in Figure 6b.

**Figure 7:**
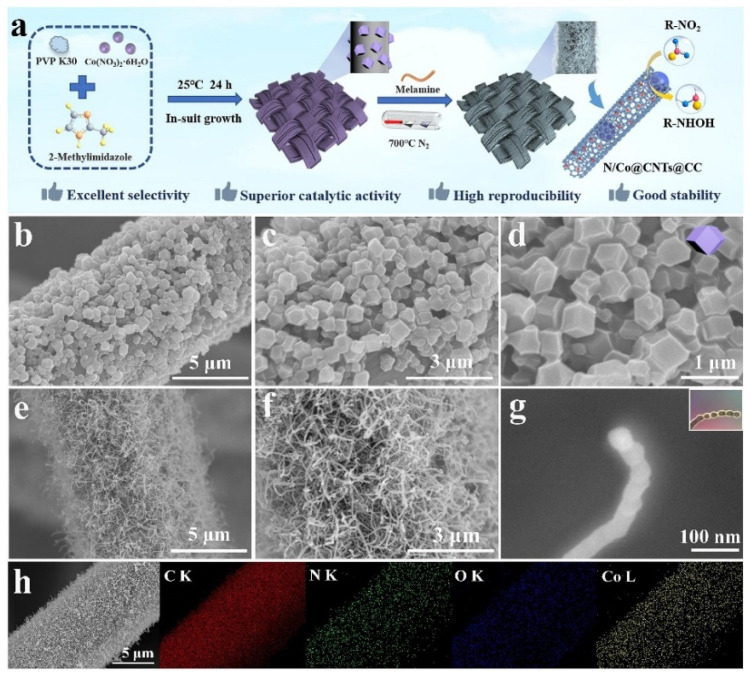
(**a**) Schematic picture shows the fabrication of N/Co@CNTs@CC composite. SEM images of ZIF-67@CC (**b**–**d**) and N/Co@CNTs@CC-II (**e**–**g**). EDX mapping results for N/Co@CNTs@CC-II (**h**). Reproduced with permission [51].

Graphite sheets (GSs) were adopted as a conductive support and poly(methylene blue) (PMB) was deposited on the GS surface to develop the NFT sensor [50]. It was believed that the presence of the PMB film may significantly improve the sensitivity of the NFT sensor. Therefore, to confirm this, a PMB-modified GS-based electrode was explored for NFT detection using the DPV method. The authors observed that the current response for NFT detection linearly increased with an increasing NFT concentration. The LD of 55 nM, sensitivity of 0.297 μA/μmol L^−1^, and LDR of 5 µM to 100 µM were observed for NFT sensing. The real-time sensing studies also displayed satisfactory recovery for NFT detection in urine and tap water samples, which indicated its strong potential for the development of flexible NFT sensors. It is well-known that ZIFs are porous materials, with abundant active sites and decent electrical conductivity. It is also believed that ZIF-67-derived metal NPs or metal oxides may exhibit better surface and conductive properties. Thus, ZIF-derived cobalt (Co) NPs were embedded into the N-doped CNTs using novel strategies, as shown in Figure 7a [51]. The obtained N/Co@CNTs@CC (CC = carbon cloth) was characterized by the SEM technique. The SEM results for ZIF-67@CC and N/Co@CNTs@CC-II are shown in Figure 7b–d and Figure 7e–g, respectively.

The SEM study revealed that ZIF-67 is grown on the surface of CC and has a typical rhombic dodecahedra shape (Figure 7b,c). Furthermore, investigations show that pyrolytic carbonization with melamine (as N-source) converted the ZIF-67 to N-doped CNTs, as shown in Figure 7e. The presence of Co NPs with N-doped CNTs were also confirmed by EDX mapping studies, as shown in Figure 7h. The N/Co@CNTs@CC-II-based NFT sensor delivered an LD of 18.41 nM and sensitivity of 8.19 μA μM^−1^ cm^−2^, with excellent recovery in milk and tap water samples. Although the above-mentioned sensor displayed reasonably good performance, it involves a high temperature of 700 °C for the material preparation. In our opinion, it is required to develop the electrode materials at a low temperature for the construction of NFT sensors. Nguyen et al. [52] reported the synthesis of an Fe/graphene/porphyrin (FGP) composite, which was further deposited on the surface of the GCE for NFT monitoring applications. The CV-based investigations revealed that the FGP-modified GCE has a better current response for NFT detection compared to the bare GCE. This may be due to the presence of synergy effects and the electrocatalytic behavior of FGP. The DPV-based studies also revealed that FGP-based GCE electrodes exhibit two LDRs of 0.5 to 50 µM and 50 µM to 200 µM for the sensing of NFT, and an LD of 246 nM. In another study [53], FeCo alloy NP confined S, N-doped bamboo-like CNTs (FeCo@S, N-CNTs) were fabricated by employing a bimetallic MOF as the precursor. The synthesized FeCo@S, N-CNTs were deposited on the SPE surface and its electrochemical sensing behavior for NFT detection was checked using CV and DPV methods. The FeCo@S, N-CNTs/SPE exhibited an LD of 3.5 nM and LDR of 0.01 µM to 75 µM for NFT sensing, with satisfactory recovery in milk, honey, egg, and chicken samples. The enhanced performance of the above-mentioned NFT sensor may be attributed to the combined effects of S, N-doped CNTs, synergy of Fe and Co, adequate active sites, rich defects, nanoconfined space, and periodic bamboo-like nodes. The proposed NFT sensor was also found to be highly selective, stable, and reproducible for NFT monitoring. The disposable nature of the FeCo@S, N-CNTs/SPE makes it a promising NFT sensor for rapid detection of NFT for practical applications.

### 3.2. Metal Oxide-Based Materials for NFT Sensing

Metal oxides are promising electrode materials for long-term stable electrochemical devices, but the presence of low conductivity is the major concern for metal oxide-based devices. It is believed that the conductivity of the metal oxides may be enhanced by incorporating them with graphene or other conductive materials. Selvi et al. [54] proposed the fabrication of a tungsten trioxide (WO_3_)-modified graphene composite for the construction of NFT sensors. It was observed that presence of graphene with WO_3_ not only improve the electrical conductivity but also enhanced the sensing performance of the graphene/WO_3_-modified SPCE for NFT detection. The graphene/WO_3_-modified SPCE exhibited an LD of 2 nM, selectivity, stability, reproducibility, and LDR of 0.01 µM to 234 µM, with appreciable recovery of NFT in biological samples. In another investigation [55], 3D NiO flowers were entrapped with B-doped g-C_3_N_4_ to form the hybrid composite electrode material for the construction of NFT electrochemical sensors. The NiO/B-g-C_3_N_4_ composite was deposited on the surface of the GCE using a simple fabrication process. The NiO/B-g-C_3_N_4_/GCE displayed good electrochemical activities for redox reactions towards the determination of NFT. The proposed NiO/B-g-C_3_N_4_/GCE delivered a low LD of 10 nM and wide LDR of 0.05 to 230 µM, with satisfactory recovery of NFT in human serum samples. The enhanced performance of the above NFT sensor may be ascribed to the presence of synergy between N ions and C_3_N_4_ matrix, high surface area, and decent conductivity. The authors proposed that the NiO/B-g-C_3_N_4_/GCE may also be utilized as an electrochemical sensor for the monitoring of NFT in clinical human health analysis, and food industries. Mariyappan et al. [56] reported that rare earth orthoferrite-based perovskite materials may be utilized as a sensing layer, and their electrochemical properties can be enhanced by combining them with conductive materials. In this context, gadolinium orthoferrite (GdFeO_3_) was combined with rGO by employing a hydrothermal-assisted synthetic method.

**Figure 8:**
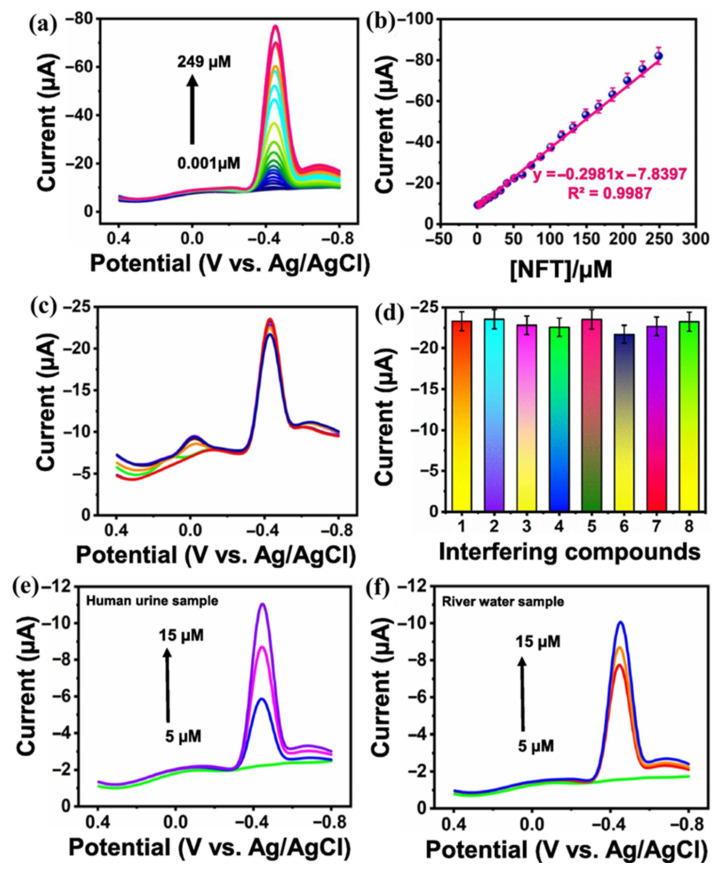
(**a**) DPV curves of the GdFeO_3_/rGO/GCE in presence of various concentrations of NFT and (**b**) corresponding calibration curve. (**c**) DPV curves and (**d**) bar graph of the GdFeO_3_/rGO/GCE for NFT detection in presence of various interfering substances. DPV responses of the GdFeO_3_/rGO/GCE for NFT detection in (**e**) human urine and (**f**) river water samples. Reproduced with permission [56].

**Figure 9:**
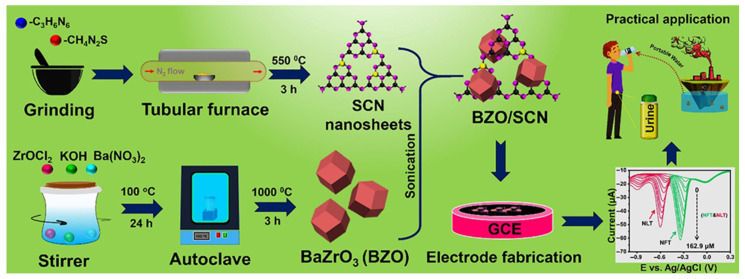
Schematic representation of the construction of BZO/S-g-C_3_N_4_ (SCN)/GCE. Reproduced with permission [58].

The synthesized GdFeO_3_/rGO composite was utilized as the sensing layer for NFT detection and the constructed electrode (GdFeO_3_/rGO/GCE) exhibits that the current response linearly increases with an increasing NFT concentration, as depicted Figure 8a,b. The proposed GdFeO_3_/rGO/GCE-based NFT sensor also demonstrated a satisfactory LD of 15.3 nM, sensitivity of 4.1985 μA μM^−1^ cm^−2^, and LDR of 0.001 to 249 μM. The authors also found that GdFeO_3_/rGO/GCE has good selectivity for NFT detection in the presence of various interfering (dopamine, 4-nitrobenzene, 4-nitro phenol, metronidazole, hydroquinone, nitrofurazone, and furazolidone) substances (Figure 8c,d), with acceptable recovery in human serum (Figure 8e) and river water (Figure 8f) samples. Nataraj et al. [57] also proposed the fabrication of a barium titanate (BaTiO_3_)/CNF composite for electrochemical sensing applications. The BaTiO_3_/CNF was prepared by employing hydrothermal and sonication methods and the formation of the desirable material was authenticated by XRD analysis, which suggested the presence of decent phase purity and the crystalline nature of the prepared composite material. The BaTiO_3_/CNF-based NFT sensor delivered an LD of 5.6 nM and LDR of 0.06 µM to 450 µM using the amperometry method. Moreover, satisfactory results for NFT detection in river water, human urine, and blood serum samples suggest its potential for practical applications. A barium zirconate (BaZrO_3_; BZO)/S-g-C_3_N_4_ (BZO/SCN) composite was also prepared using the hydrothermal method followed by the sonication method (Figure 9) [58]. The synthesized material BZO/SCN was coated on the GCE, and its electrochemical properties were evaluated by the DPV technique. The BZO/SCN/GCE exhibits an LD of 2 nM, LDR of 0.09 µM to 260.9 µM, high selectivity, repeatability, stability, reproducibility, and a sensitivity of 35.73 µA µM^−1^ cm^2^ for NFT detection using the DPV technique.

Sudha et al. [59] reported a novel coriander leaf-shaped scandium molybdate (Sc_2_Mo_3_O_12_ = ScMo)-decorated f-MWCNT composite via ultrasonication treatment. The obtained ScMo@f-MWCNT composite has a high surface area and good electrical conductivity. Thus, these authors modified the surface of the GCE with the ScMo@f-MWCNTs electrocatalyst and used it as an NFT sensor. This constructed NFT sensor (ScMo@f-MWCNTs/GCE) demonstrated a wide LDR of 0.1 to 180 µM, LD of 9.3 nM, sensitivity of 0.5136 μA μM^−1^cm^−2^, decent storage stability, repeatability, reproducibility, and selectivity for NFT monitoring. These satisfactory results for real sample studies in biological samples suggest that the ScMo@f-MWCNTs/GCE is a promising electrode for NFT sensing. Li et al. [60] also found that molybdenum disulfide (MoS_2_) is a layered 2D material with excellent electrochemical and acceptable conductivity. The combination of MoS_2_ with other material may form heterojunctions and the presence of synergy effects may further improve electrochemical performance of the MoS_2_-based sensors. The cobalt molybdate (Co_2_Mo_3_O_8_)/MoS_2_ hybrid composite was fabricated on CC and its electrochemical features were evaluated for NFT sensing using voltammetry methods. The Co_2_Mo_3_O_8_/MoS_2_/CC demonstrated excellent electrochemical properties for NFT detection, which may be attributed to the high redox response, abundant defect sites, fast electron transport process, and enhanced electrical conductivity. Therefore, Co_2_Mo_3_O_8_/MoS_2_/CC successfully detected NFT in honey, milk, and tap water samples with satisfactory recovery. Babulal et al. [61] proposed the construction of samarium vanadate (SV) NPs using the co-precipitation method followed by a sintering process. The sintering temperature for the preparation of SV NPs was 450, 550, and 650 °C, and obtained samples were labeled as SV-1, SV-2, and SV-3, respectively. Furthermore, SV NPs were combined with graphene sheets via a sonication approach and labelled as SVG-1, SVG-2, and SVG-3. It was found that the SVG-2-modified GCE has improved electrochemical properties due to the synergistic interactions between SV-2 and graphene. The SVG-2/GCE delivered an LD of 8.7 nM, LDR of 0.035 µM to 672.3 µM, and sensitivity of 0.875 μA mA^−1^ cm^−2^, with satisfactory recovery in blood serum and human urine samples. Sridharan et al. [62] reported the facile preparation of iron oxide and h-BN using the hydrothermal method. The obtained α-Fe_2_O_3_/h-BN-based electrode exhibited an LDR of 0.025 µM to 22.95 µM, sensitivity of 2.36 µA µM^−1^ cm^−2^, and LD of 15 nM for NFT detection. Alagumalai et al. [63] proposed that a tin bismuth oxide (SnBi_2_O_3_)/GO composite may exhibit improved electrochemical properties for the development of electrochemical sensors. The obtained SnBi_2_O_3_/GO exhibits acceptable crystallinity, and the presence of SnBi_2_O_3_ on the GO surface was confirmed by SEM analysis. Furthermore, SnBi_2_O_3_/GO was coated on the GCE surface using a simple fabrication procedure. The fabricated SnBi_2_O_3_/GO/GCE has the potential to detect the NFT with an LD of 12.4 nM and sensitivity of 2.857 μA μM^−1^ cm^−2^. In addition, this NFT sensor has a good LDR of 0.023 μM to 814.36 μM, high selectivity in the presence of interfering substances, and satisfactory recovery of NFT in water and biofluids samples. Kumar et al. [64] reported a novel 3D flower-shaped neodymium molybdate (Nd_2_Mo_3_O_9_) by employing the sol–gel method. The obtained Nd_2_Mo_3_O_9_ was deposited on the SPCE surface and its electrochemical activity for NFT detection was evaluated by the DPV technique. The authors were able to obtain the LD of 16 nM, LDR of 0.1 µM to 21 µM and 28 µM to 481 µM, with excellent selectivity in the presence of various interfering compounds.

### 3.3. Polymers/Metal Sulfides/LDH/MXenes

As per recent studies, it was found that polymers, MXenes, and layered double hydroxide (LDH) materials are also promising sensing materials due to their conductive nature and electrochemical properties. Dechtrirat et al. [65] reported the fabrication of an MIP and Au NP/poly(3, 4-ethylenedioxythiophene)/poly(styrene sulfonate)-based SPCE for the determination of NFT using the DPV technique. It was also found that the Au NP/PEDOT/PSS and MIP-based electrode has improved catalytic properties and conductivity. Thus, an LD of 0.1 nM, LDR of 1 nM to 1000 nM, and stability of 45 days were observed for the monitoring of NFT. The presence of conductive PEDOT/PSS and synergy effects of the composite material enhanced the detection of NFT in real samples and suggest its promising role for practical applications. Vinothkumar et al. [66] combined g-C_3_N_4_ and PPy for the construction of a g-C_3_N_4_/PPy-based electrode for the determination of NFT. The prepared g-C_3_N_4_/PPy also exhibits an improved surface area, conductivity, and synergism, which enhanced the sensing performance of the developed NFT sensor. The sensitivity of 7.813 µA µM^–1^ cm^–2^, LD of 5 nM, and LDR of 0.4 µM to 585.2 µM were obtained for NFT detection using voltammetric methods. Vilian et al. [67] constructed a Au NP/PPy/titanium carbide (Ti_3_C_2_T_x_) composite by employing the sonochemical route and oxidant-free polymerization methods. The synthesized Au NP/PPy/Ti_3_C_2_T_x_ composite was coated on the GCE surface and EIS studies revealed that the Au NP/PPy/Ti_3_C_2_T_x_/GCE has an electron transfer rate content value of 1.03 × 10^−2^ cm/s, with a low charge transfer resistance value (Rct) of 73 Ω. This revealed the presence of improved electrical conductivity and decent electrocatalytic properties in the prepared Au NP/PPy/Ti_3_C_2_T_x_ composite. Therefore, Au NP/PPy/Ti_3_C_2_T_x_/GCE delivered excellent recovery of NFT pond water, honey, and hospital wastewater samples. Additionally, these authors obtained an LD of 0.26 nM and LDR of 6 nM to 172 nM, with decent selectivity for NFT detection. Tumrani et al. [68] reported the formation of palladium (Pd) nanocubes (NCs) embedded with partially oxidized Ti_3_C_2_T*_x_*-TiO_2_. The authors observed that the proposed electrocatalyst exhibited an LD of 0.01 nM and LDR of 1 nM to 140 nM, with a decent selective nature for NFT detection. The authors proposed that the presented sensor may be explored for the sensing of NFT in pharmaceutical and environmental monitoring applications. Shui et al. [69] utilized novel strategies for a highly selective and sensitive NFT sensor. In this context, a polydopamine (PDA)-derived C coated NiCo@C and FeCo@C nanohybrid (NiCo@C/FeCo@C@C) was proposed as the sensing material, which displayed good selectivity and sensitivity for NFT detection. This sensor can detect NFT with an LDR of 0.05 to 100 µM and LD of 14 nM, with high reproducibility and long-term stability. This enhanced electrochemical activity for NFT detection may be ascribed to the synergism and high surface area of the electrode material. Liu et al. [70] also explored a one-step electrodeposition approach for the formation of a Ru/NiFe-LDH-MXene composite and deposited it on the SPCE surface. The Ru/NiFe-LDH-MXene/SPCE demonstrated a high sensitivity of 152.44 μA μM^−1^ cm^−2^ and LD of 2.2 nM, which may be due to the synergistic interactions between the 2D LDH and MXene material and catalytic properties of Ru NPs. Liu et al. [71] stated that 2D/2D van der Waals heterojunction of vanadium disulfide (VS_2_) and Ti_3_C_2_T_x_ MXene may enhance the electrochemical determination of NFT. The VS_2_/Ti_3_C_2_T_x_-modified SPCE shows an LD of 4.7 nM and LDR of 0.01 µM to 400 µM, with strong resistance to the interfering substances. The presence of synergy and good conductivity of the MXene-based composite enhanced the electrochemical determination of NFT.

### 3.4. Other Materials for NFT Sensing

Roushani et al. [72] reported an imprinted molecular sensor for the sensing of NFT, which demonstrated an LD of 0.3 nM and LDR of 0.001 µM to 0.05 µM and 0.1 µM to 1 µM, with satisfactory selectivity towards the detection of NFT. In another study [73], a β-cyclodextrin (β-CD)/CNF composite was also utilized as the electrochemical sensing material, which delivered an LD of 1.8 nM and LDR of 0.004 µM to 308 µM, with decent stability and selectivity. This sensor also shows satisfactory NFT recovery in spiked urine and blood samples. As per the study of Ezazi et al. [74], it was proposed that a Ag/Ni(OH)_2_ composite can be used as the sensing layer for the monitoring of NFT. Therefore, Ag/Ni(OH)_2_ was coated on the GCE surface and its sensing behavior was checked by employing the DPV technique. The proposed sensor was found to be efficient for the monitoring of NFT, with an LDR of 0.11 µM to 13 µM and 13 µM to 212 µM. Adane et al. [75] prepared a novel composite of a Au-Ag alloy nanocoral cluster/zinc oxide NP/CPE/polyethylene oxide (Au-Ag-ANCCs/ZnO-NPs-CPE/PEO) using simple strategies. The Au-Ag-ANCC/ZnO-NP-CPE/PEO was found to be highly selective for NFT detection, with an LD of 0.26 pM and LDR of 1 pM to 250 µM. In addition, the proposed sensor displayed acceptable reproducibility, selectivity, repeatability, and stability. The above aforementioned reports show that the electrochemical method is a promising technology for the construction of NFT sensors. Electrochemical activity of the various electrodes for NFT sensing are summarized in Table 2.

## 4. Conclusions and Perspectives

In conclusion, it is worth mentioning that significant progress has been made towards the development of electrochemical sensors for the determination of NFZ and NFT. Various advanced materials such as carbon-based materials (graphene, carbon nanotubes, graphitic carbon nitride, etc.), metal oxide, Au/Ag NP, polymer, LDH, MOF, and MXene-based hybrid composites were explored as electrocatalysts. The observations show that Au NP/rGO/MIP-modified GCEs exhibited an excellent detection limit (0.00018 µM) for NFZ detection, whereas SnSe/h-BN-modified electrodes demonstrated a detection limit of 0.00034 µM. The Au NP-based electrodes are expensive for practical applications. SnSe/h-BN-based electrodes may suffer from the presence of the toxic nature of Se. In contrast, Ru/NiFe-LDH-MXene/SPCEs exhibited a detection limit of 0.0022 µM for the sensing of NFT. MXene and MOF-based materials may be promising electrode modifiers for the construction of NFZ and NFT sensors. Despite their excellent and promising selectivity, low detection limit, and sensitivity, some challenges exist, which need to be overcome.

- MXene-based materials are promising next-generation electrode materials, but have major concerns such as harsh conditions of HF for etching treatments.
- MOF-based materials have a larger surface area with high porosity, but their limited conductivity is one of the major concerns.
- The development of cost-effective and environmental friendly methods are of great significance to fabricate the novel hybrid electrode materials.
- LDH/Mene and MOF-based hybrid composites should be optimized for NFZ and NFT sensing applications.
- The long-term stability, real-time monitoring, and selectivity should be improved for practical applications.
- The depth mechanism for NFZ and NFT should be studied on MXene-based materials.
- The simultaneous detection of NFZ and NFT with appropriate differentiation should be studied.
- The fabricated NFZ and NFT sensors should be miniaturized and incorporated into portable devices.

## Figures and Tables

**Table 1:** Electrochemical activities of various electrode materials for NFZ sensing.

Materials/Electrode	Synthesis Method	LD (µM)	LDR (µM)	Sensitivity	Detection technique	Real Sample	References
Hollow MIL-101/GCE	Crystal growth/acid etching	0.01	0.030 to 55	-	DPV	Milk, honey, and crayfish	[21]
DUT-67/T-PPY-2/GCE	In situ growth	8.7	9.08 to 354.08; 354.08 to 1004.04	0.175 μA/μM	DPV	Milk and lake water	[22]
BC/Cr_2_O_3_/Ag/MIP/GCE	Precipitation polymerization/sonication	0.003	0.005 to 10	-	DPV	Blood and urine	[23]
AuNPs/UiO-66-NH_2_/SPCE	Solvothermal	0.003	0.01 to 50	-	DPV	Lake water	[24]
2D-Co MOF/GCE	Slow evaporation (80 °C; 5 days)	0.04	0 to 25	-	SWV	Food and biological samples	[25]
MWCNTs/COOH/GCE	Mixed acid phase oxidation	0.224	-	-	AMP (i-t)	Pork liver sample	[26]
[Ru-PMo_12_/PDDA-GO]_3_	Electrodeposition	0.08952	-	-	Chronoamperometry	Crayfish	[28]
Au NPs/rGO/MIP	Electropolymerization	0.00018	0.005 to 1	-	DPV	Milk	[29]
LIG-50 array	Laser engraving technique	0.035	0.2 to 8	-	LSV	Fish meat	[30]
TiO_2_/Au-NFs/O-C_3_N_4_/GCE	Solvothermal	0.001	0.008 to 105	1.40 µA/µM.cm^2^	AMP	Human urine and river water	[31]
g-C_3_N_4_/Gd_2_MoO_6_/GCE	Co-precipitation/ultrasonication	0.006	0.02 to 2000	2.057 µA/µM.cm^2^	DPV	Milk and human urine	[32]
Sg–C_3_N_4_/CuWO_4_/GCE	Hydrothermal/sonication	0.003	0.005 to 52.305	1.24 µA/µM.cm^2^	DPV	Urine and blood serum samples	[33]
NdO@TC/GCE	Ultrasonication	0.0027	0.01 to 2231	0.1914 µA/µM.cm^2^	DPV	Urine, blood serum, and tap water	[34]
FeTiO_3_/GCE	Hydrothermal	0.002	0.01 to 162.2	0.551 µA/µM.cm^2^	-	-	[35]
DFO/S-g-C_3_N_4_/GCE	Reflux/sonication	0.0071	0.025 to 180.13	2.929 µA/µM.cm^2^	AMP (i-t)	Lake and river water	[36]
Crab shell (CS)/GCE	High temperature heating	0.11	0.40 to 80	0.55 μA/μM	DPV	Compound cod liver ointment	[37]
Au-AuNR	Sonoelectrodeposition	0.18	3 to 500	-	DPV	Urine and blood serum	[38]
Ag-NPs@CPE	-	0.01	-	-	-	Urine and tap water	[39]
Au-Ag modified electrode	Electrodeposition	0.2	1.99 to 643.49	-	-	-	[40]
Ag-NPs@CPE	Calcination	0.012	0.2 to 100	-	DPV	Urine, tap water and milk	[42]
SnSe/h-BN/GCE	Solvothermal	0.00034	0.001 to 12.12; 15.2 to 342.2	1.927 µA/µM.cm^2^	AMP (i-t)	Urine and water	[43]
NMCN-CPE	Hydrothermal	4.3	30 to 570	-	AMP	Ointments	[44]

**Table 2:** Electrochemical performance of various electrode materials for NFT sensing.

Materials	Synthesis Method	LD (µM)	LDR (µM)	Sensitivity	Detection Technique	Real Sample	References
HA NPs/MWCNT-CHI/GCE	Hydrothermal/ultrasonication	0.0013	0.005 to 982.1	-	AMP	Water and pharmaceutical tablet	[47]
NiFe/*f*-MWCNT/SPCE	Hydrothermal/ultrasonication	0.03	-	11.45 µA/µM.cm^2^	DPV	Human serum and urine	[48]
f-CNF/CB/GCE	Sonication	0.016	0.05 to 104.66	15.25 µA/µM.cm^2^	AMP	-	[49]
GS/PMB	Electropolymerization	0.055	5 to 100	0.297 µA/µM	DPV	Urine and tap water	[50]
N/Co@CNTs@CC-II	in-situ growth and sublimation-gas phase	0.01841	-	8.19 µA/µM.cm^2^	LSV	Milk and tap water	[51]
Fe/Graphene/Porphyrin/GCE	Self-assembled method	0.246	0.5 to 200	0.9808 µA/µM.cm^2^	DPV	Milk	[52]
FeCo@S,N-CNTs	Thermal treatment	0.0035	0.01 to 75.0	-	DPV	Honey, egg, fish, chicken, and milk	[53]
Gr/WO_3_/SPCE	Calcination	0.002	0.01 to 234	2.18 µA/µM.cm^2^	LSV	Urine and tap water	[54]
NiO/BCN	Polycondensation/hydrothermal	0.01	0.05 to 230	-	AMP	Urine	[55]
GdFeO_3_/RGO	Hydrothermal	0.0153	0.001 to 249	4.1985 µA/µM.cm^2^	DPV	Urine and river water	[56]
BT/CNF	Hydrothermal	0.005	0.06 to 450	1.76 µA/µM.cm^2^	AMP (i-t)	Urine, river water, and blood serum	[57]
BZO/SCN/GCE	Hydrothermal/sonication	0.002	0.09 to 260.9	35.73 µA/µM.cm^2^	DPV	Water	[58]
ScMo@*f*-MCN Ncs/GCE	Ultrasonication	0.0093	0.01 to 180	0.5136 µA/µM.cm^2^	DPV	Blood serum, urine, and lake water	[59]
Co_2_Mo_3_O_8_/MoS_2_@CC	Hydrothermal	0.0119	100 to 700	27.6 µA/µM.cm^2^	LSV	Milk, honey, and tap water	[60]
SVG-2/GCE	Co-precipitation/Ultrasonication	0.0087	0.0035 to 672.3	-	AMP (i-t)	Blood serum and urine	[61]
α-Fe_2_O_3_/h-BN	Hydrothermal	0.015	0.025 to 22.95	2.36 µA/µM.cm^2^	AMP	Urine	[62]
SnBi_2_O_3_/GO/GCE	Hydrothermal/sonication	0.0124	0.023 to 814.36	2.857 µA/µM.cm^2^	DPV	Urine and tap water	[63]
NdM/SPCE	Sol-gel	0.016	0.1 to 21; 28 to 481	µA/µM.cm^2^	DPV	River water, lake water, and urine	[64]
g-C_3_N_4_/PPy	Sonochemical	0.005	0.04 to 585.2	7.813 µA/µM.cm^2^	DPV	Urine and blood serum	[66]
Au-PPy-MXene-GCE	Sonication	0.00026	6 to 172	-	AMP	Pond water, honey, and hospital wastewater	[67]
Pd–Ti_3_C_2_T*_x_*–P	-	0.00001	0.001 to 0.14	-	DPV/AMP	Urine and hospital waste effluent	[68]
NiCo@C/FeCo@C@C/GCE	Polymerization/pyrolysis	0.014	0.05 to 100	-	DPV	Lake water	[69]
Ru/NiFe-LDH-MXene/SPCE	Electrodeposition	0.0022	0.01 to 275	152.44 µA/µM.cm^2^	LSV	Honey and milk	[70]
VS_2_/Ti_3_C_2_T_x_/SPCE	Liquid phase mixing/annealing	0.0047	0.01 to 400	-	LSV	Milk, lake water, honey, and tap water	[71]
β-CD/CNF	Ultrasonication	0.0018	0.004 to 308	-	AMP	Blood serum, tablet, and urine sample	[73]

## Data Availability

Data sharing is not possible. No new data were generated.
